# gp96与免疫相关基因*CTLA-4*、*CD8*在肺癌组织芯片中的表达及意义

**DOI:** 10.3779/j.issn.1009-3419.2010.08.08

**Published:** 2010-08-20

**Authors:** 海燕 郑, 赟 李, 兴芬 王, 向莲 张, 新允 王

**Affiliations:** 1 300211 天津，天津医科大学第二医院病理科 Department of Pathology, the Second Hospital Affilated to Tianjin Medical University, Tianjin 300211, China; 2 300070 天津，天津医科大学病理学教研室 Department of Pathology, Tianjin Medical University, Tianjin 300070, China

**Keywords:** 肺肿瘤, 组织芯片, gp96, CTLA-4, CD8, 免疫组织化学, Lung neoplasms, Tissue microarray, gp96, CTLA-4, CD8, Immunohistochemistry

## Abstract

**背景与目的:**

近年来研究显示gp96在机体发挥抗肿瘤的特异性细胞毒免疫应答中发挥重要作用。本研究旨在探讨热休克蛋白gp96与免疫相关基因*CTLA-4*、*CD8*在不同进展阶段肺癌中的表达及生物学意义。

**方法:**

应用免疫组化Envision法及组织芯片技术检测89例原发肺癌、12例癌前病变、10例正常肺组织及12例淋巴结转移性肺癌中gp96、CTLA-4、CD8的表达，并分析其与肺癌临床病理指标的关系。

**结果:**

① 原发性肺癌组gp96的阳性率高于正常组与癌前病变组（*P* < 0.05）。原发性肺癌组和癌前病变组CTLA-4的阳性率均高于正常组（*P* < 0.05）。CD8在原发性肺癌组的阳性率高于正常组（*P* < 0.05）。CD8阳性淋巴细胞高表达组gp96阳性率低于低表达组（*P* < 0.05）。②gp96表达与患者的性别、分化程度和临床分期有关（*P* < 0.05），而与年龄、肉眼类型、组织学分型和有无淋巴结转移无关（*P* > 0.05）。CTLA-4表达与年龄、分化程度有关（*P* < 0.05），而与性别、肉眼类型、组织学分型、临床分期和有无淋巴结转移无关（*P* > 0.05）。CD8表达与临床分期有关（*P* < 0.05），而与性别、年龄、肉眼类型、组织学分型、分化程度和有无淋巴结转移无关（*P* > 0.05）。在鳞癌和小细胞癌中，gp96、CTLA-4的表达阳性率均高于CD8的表达（*P* < 0.05）。③Gp96与CTLA-4表达呈正相关（*P* < 0.05），与CD8表达呈负相关（*P* < 0.05），CD8与CTLA-4表达呈负相关（*P* < 0.05）。

**结论:**

在肺癌中gp96的表达与CTLA-4、CD8密切相关，提示三者在肺癌的发生发展中起重要作用，可作为评估患者病情和估计预后的参考指标。

## Abstract (en)

**Background and objective:**

It has been proven that gp96 plays an important role in specific cytotoxic immune response which is involved in anti-tumor effect in the body. The aim of this study is to investigate the biological significance of heat shock protein gp96 and immune-related gene *CTLA-4*, *CD8* expressions in lung cancer tissues of different progressive stages.

**Methods:**

We used Envision immunohistochemistry method to detect the levels of expression of gp96, CTLA-4, CD8 in tissue microarray, which contained 89 primary lung cancer tissues, 12 lymph node metastasis lung cancer tissues, 12 precancerous lesions and 10 normal lung tissues, and analyzed the relationship between their expressions and clinicopathological parameters.

**Results:**

(1) The positive rate of gp96 in primary lung cancer was remarkably higher than that in precancerous lesion and normal lung tissue (*P* < 0.05). The positive rate of CTLA-4 in primary lung cancer tissue and precancerous lesion was significantly higher than that in normal lung tissue (*P* < 0.05). The positive rate of CD8 in primary lung cancer tissue was significantly higher than that in normal lung tissue (*P* < 0.05). The positive rate of gp96 in CD8-positive lymphocytes in the high expression group was less than that in the low group (*P* < 0.05). (2) The positive rate of gp96 was closely related to sex, differentiation and clinical stage (*P* < 0.05), but not to age, gross type, histological type and lymph node metastasis (*P* > 0.05). The positive rate of CTLA-4 was closely related to age and differentiation (*P* < 0.05), but not to sex, gross type, histological type, clinical stage and lymph node metastasis (*P* > 0.05). CD8 expression was related to clinical stage (*P* < 0.05), but not to sex, age, gross type, histological type, differentiation and lymph node metastasis (*P* > 0.05). The positive rates of gp96, CTLA-4 were higher than that of CD8 in squamous cell carcinoma and SCLC, respectively. (3) There was positive correlation between gp96 and CTLA-4; there was negative correlation between gp96 and CD8, and there was negative correlation between CD8 and CTLA-4 (*P* < 0.05).

**Conclusion:**

Gene expression of PD-L1 on lung cancer cell line can decrease the cytolytic effect of CTL on target cells.

肺肿瘤尤其是肺癌发病率不断上升，其发病率和死亡率在男性恶性肿瘤中占第一位，在女性恶性肿瘤中占第二位，且随年龄增长而上升。热休克蛋白（heat shock protein, HSP）又称应激蛋白，gp96是HSP家族成员之一，可作为分子伴侣参与其它蛋白质的折叠、转运、合成等过程，国内外研究发现许多肿瘤细胞表面可以高度表达或特异表达不同亚族的HSP，在机体发挥抗肿瘤的特异性细胞毒免疫应答中起重要作用。CD8是细胞毒T淋巴细胞，可抑制抗体合成、分泌并抑制T细胞的增殖，改变机体的免疫状态。细胞毒性T淋巴细胞相关抗原4（cytotoxic T lymphocyte associated antigen-4, CTLA-4）是一种免疫调节分子，可通过降低T细胞活性抑制机体的抗肿瘤免疫反应。本研究采用免疫组化Envision法及组织芯片技术检测gp96、CTLA-4、CD8在肺癌中的表达，探讨gp96与免疫相关基因的关系，为患者治疗方案提供一个新的参考指标。

## 1 材料与方法

### 1.1 临床资料

收集天津医科大学总医院及天津医科大学第二医院病理科1987年1月-2003年12月手术切除的肺癌石蜡包埋标本制作组织芯片，其中原发肺癌89例，淋巴结转移性肺癌12例，肺癌癌前病变12例。患者术前均未行放化疗。按照世界卫生组织肺及胸膜肿瘤组织学类型修订方案^[1]^，并参考《外科病理诊断学》^[2]^对全部资料进行统一的命名和分类。原发肺癌中包括腺癌35例，鳞癌33例，小细胞癌12例，大细胞癌9例; 男性67例，女性22例; 年龄33岁-78岁，平均年龄（60.28±9.48）岁; 按肉眼类型：周围型59例，中央型30例; 按组织学分级：高+中分化53例，低+未分化36例; 按临床分期：Ⅰ期+Ⅱ期56例，Ⅲ期+Ⅳ期33例; 有淋巴结转移者48例，无淋巴结转移者41例。正常肺组织10例，包括手术切除炎性假瘤病例的正常肺组织3例、尸检的正常肺组织6例以及肺大泡的正常肺组织1例。

### 1.2 研究方法

组织芯片共计270点，包括实验组原发性肺癌89例，淋巴结转移性肺癌12例，肺癌癌前病变12例，对照组正常肺组织10例。正常肺组织和原发肺癌每例取2点，癌前病变和淋巴结转移性肺癌每例取3点。采用免疫组化Envision法，肿瘤组织切片按抗体说明书进行高压热修复，单克隆抗HSPgp96抗体（BIOS公司）、CTLA-4（BIOS公司）、CD8（北京中杉金桥生物技术有限公司）标记，DAB显色、苏木素复染，每批染色均设阳性对照，PBS取代一抗作阴性对照。

### 1.3 免疫组化着色结果判断

gp96阳性表达定位于癌细胞胞浆（图 1A），CTLA-4阳性表达定位于癌细胞、淋巴细胞胞浆（图 1B），CD8阳性表达定位于淋巴细胞胞膜（图 1C）。结果判断标准参考有关文献^[3]^，按着色强度分为：0分为无色，1分为浅棕黄色，2分为棕黄色，3分为棕褐色; 再将阳性细胞所占的百分比打分，1分为 < 10%，2分为10%-50%，3分为51%-75%，4分为 > 75%，根据二者乘积判断阳性等级： < 3分为阴性，> 3分为阳性。

**1:**
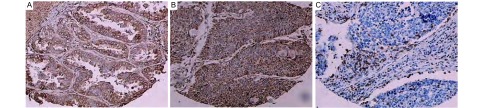
肺腺癌、肺鳞癌以及肺小细胞癌淋巴细胞组织中gp96、CTLA-4、CD8的表达（Envision法，×100）。A：肺腺癌组织gp96的表达; B：肺鳞癌组织CTLA-4的表达; C：肺小细胞癌淋巴细胞CD8表达。 gp96、CTLA-4、CD8 expressions in adenocarcinoma, squamous cell carcinoma, lymphocyte of small cell lung cancer (Envision method, ×100). A: gp96 positive expression in adenocarcinoma; B: CTLA-4 positive expression in squamous cell carcinoma; C: CD8 positive expression in lymphocyte of small cell lung cancer.

### 1.4 统计学处理

采用SPSS 11.5统计软件包进行数据分析，gp96、CTLA-4、CD8在各组中表达阳性率的比较采用行×列表，进行χ^2^检验或*Fisher*确切概率法计算; 各指标间表达强度的相关关系采用*Spearman*等级相关分析。以*P* < 0.05为差异具有统计学意义。

## 2 结果

### 2.1 gp96、CTLA-4和CD8在肺癌进展组织芯片中的表达情况

gp96在正常组、癌前病变组、原发性肺癌组、淋巴结转移性肺癌组的表达阳性率分别为20.00%、58.33%、87.64%、91.67%，原发性肺癌组阳性率高于正常组与癌前病变组（*P* < 0.05）。CTLA-4在正常组、癌前病变组、原发性肺癌组、淋巴结转移性肺癌组的表达阳性率分别为10.00%、83.33%、86.52%、91.66%，原发性肺癌组和癌前病变组的阳性率均高于正常组（*P* < 0.05）。CD8仅在正常组、癌前病变组、原发性肺癌组、淋巴结转移性肺癌组的间质淋巴细胞中表达，阳性率分别为0、33.33%、56.18%、75.00%，原发性肺癌组的阳性率高于正常组（*P* < 0.05）。

原发性肺癌中，根据CD8表达情况分成CD8阳性淋巴细胞高表达组和低表达组，gp96在高表达组阳性率为63.16%，低表达组阳性率为90.32%，两组间差异具有统计学意义（χ^2^=5.433, *P* < 0.05）; CTLA-4在高表达组阳性率为89.47%，低表达组阳性率为74.19%，两组间差异无统计学意义（χ^2^=1.719, *P* > 0.05）。

### 2.2 gp96、CTLA-4和CD8在原发性肺癌中的表达与临床病理参数的关系

gp96的表达与肺癌患者性别、分化程度、临床分期有关，男性表达阳性率高于女性，低+未分化组高于高+中分化组，Ⅲ期+Ⅳ期高于Ⅰ期+Ⅱ期，差异具有统计学意义（*P* < 0.05）; 与年龄、肉眼类型、组织学类型、有无淋巴结转移无关。CTLA-4的表达与肺癌患者年龄、分化程度有关，年龄≥60岁组阳性率高于 < 60岁组，低+未分化组高于高+中分化组，差异具有统计学意义（*P* < 0.05）; 与性别、肉眼类型、组织学类型、临床分期、有无淋巴结转移无关。CD8的表达与临床分期有关，Ⅲ期+Ⅳ期低于Ⅰ期+Ⅱ期，差异具有统计学意义（*P* < 0.05）; 与性别、年龄、肉眼类型、组织学类型、分化程度、有无淋巴结转移无关。鳞癌中gp96、CTLA-4表达阳性率均高于CD8的表达（χ^2^=11.000, *P* < 0.05; χ^2^=11.000, *P* < 0.05）; 小细胞肺癌中gp96、CTLA-4表达阳性率均高于CD8的表达（χ^2^=10.971, *P* < 0.05; χ^2^=8.224, *P* < 0.05）。腺癌和大细胞癌中gp96、CTLA-4、CD8之间表达差异无统计学意义（*P* > 0.05）。具体数据见表 1。

**1:** gp96、CTLA-4和CD8在原发性肺癌中的表达与临床病理参数的关系 Associations between the expressions of gp96, CTLA-4 and CD8 in lung cancer and clinicopathologic paramenters

Group	*n*	gp96				CTLA-4				CD8		
		Positive rate (%)	*X*^2^	*P*		Positive rate (%)	*X*^2^	*P*		Positive rate (%)	*X*^2^	*P*
Gender			10.215	0.001			0.553	0.457			0.101	0.751
Male	67	94.00				88.06				55.22		
Famale	22	68.20				81.81				19.40		
Age			2.801	0.094			3.958	0.047			0.597	0.440
≥60	53	92.50				92.45				52.83		
< 60	36	80.60				77.78				61.11		
Gross type		0.040	0.842			3.393	0.531			2.022	0.155
Peripheral type	59	88.10				88.14				50.85		
Central type	30	86.70				83.33				66.67		
Histological type											
Non-small cell lung caner	77	81.81	1.257	0.739		87.01	1.259	0.739		61.03	6.959	0.073
Adenocarcinoma	35	82.86				85.71				68.57		
Squamous cell carcinoma	33	90.90				90.91				54.55		
Large cell carcinoma	9	89.00				77.78				55.56		
Small cell lung caner	12	92.00				83.33				25.00		
Differentiation		5.124	0.024			5.939	0.015			1.970	0.160
High-moderate	53	81.13				79.24				62.26		
Low-undifferentiation	36	97.22				97.22				47.22		
Stage			4.214	0.040			0.867	0.352			4.031	0.045
Ⅰ+Ⅱ	56	82.14				83.93				64.28		
Ⅲ+Ⅳ	33	96.97				90.91				42.42		
Lymph node metastasis			1.559	0.212			0.840	0.359			0.760	0.383
Present	48	91.67				89.58				60.42		
Absent	41	82.93				82.93				51.22		

### 2.3 原发性肺癌中gp96、CTLA-4和CD8的相关性分析

经*Spearman*等级相关分析，gp96与CTLA-4在肺癌组织中的表达呈正相关（*rs*=0.551, *P* < 0.001），gp96与CD8表达呈负相关（*rs*=-0.263, *P*=0.013），CD8与CTLA-4表达呈负相关（*rs*=-0.216, *P*=0.042）。具体数据见表 2。

**2:** 原发性肺癌中gp96、CD8和CTLA-4的相关关系 The correlation among gp96, CD8 and CTLA-4 in primary lung cancer

		CD8			CTLA-4			Total
		+	-		+	-		
Gp96	+	40	38		73	5		78
	-	10	1		4	7		11
CD8	+	-	-		40	10		50
	-	-	-		37	2		39
Total	50	39	77		12			89

## 3 讨论

肿瘤的发生发展是一个多因素、多步骤的过程，涉及多种基因突变的积累，也是一个量变到质变的过程。HSP家族是一类极为保守的蛋白质，当机体受到有害因素（如炎症、紫外线、应急、肿瘤发生等）刺激时，细胞内HSP合成增加，它能保护机体（或细胞）不受或少受损伤。肿瘤细胞自身与机体不相协调的增生和恶性特征在一定程度上与HSP的表达密切相关：一方面作为“分子伴侣”参与肿瘤细胞的功能代谢，保护肿瘤细胞免受有害因素的损害; 另一方面由于肿瘤细胞与机体正常细胞生长、代谢、分化的异质性，使HSP成为与其相结合的肿瘤抗原多肽的靶载体，在参与肿瘤抗原的免疫反应中发挥举足轻重的作用。HSPgp96是HSP90家族的成员，它通过细胞信号转导、细胞周期调控等多种途径参与细胞的生长和增殖。研究^[4-6]^报道在胃癌、食管癌、黑色素瘤中gp96的表达增高。本研究发现gp96在正常组、癌前病变组、原发性肺癌组、淋巴结转移性肺癌组的阳性率逐步增高，且原发性肺癌组与正常组之间、原发性肺癌组与癌前病变组之间差异均具有统计学意义（*P* < 0.05），提示肿瘤环境下gp96表达增强，是局部缺血缺氧、酸中毒、葡萄糖消耗增多的应激表现，这些应激表现促进了肿瘤细胞的增殖。同时研究发现，gp96在CD8阳性淋巴细胞高表达组阳性率低于低表达组（*P* < 0.05），CD8阳性淋巴细胞浸润越多，gp96表达越低，提示gp96的表达与CD8阳性淋巴细胞浸润程度呈负相关。gp96在男性中表达阳性率高于女性（*P* < 0.05），低+未分化组高于高+中分化组（*P* < 0.05），Ⅲ期+Ⅳ期高于Ⅰ期+Ⅱ期（*P* < 0.05），与文献^[7]^报道一致。研究结果说明随肿瘤进展gp96表达升高，提示gp96的表达与肺癌的发生发展密切相关，其表达水平可以作为癌细胞调整其抗原特性以逃避免疫监视、适应生存的一种危险信号，gp96也可作为判断肿瘤分化及预后的参考指标。

恶性肿瘤患者普遍存在免疫抑制，其主要原因是免疫系统不能有效识别、排斥和消灭肿瘤细胞。机体抗肿瘤免疫的主要方式是细胞免疫，T淋巴细胞是一类介导细胞免疫功能的免疫活性细胞，CD8^+^T细胞不仅是细胞毒T淋巴细胞，也是具有免疫抑制作用的调节性T细胞。研究^[8]^证明CD8^+^T细胞具有CD28^+^和CD28^-^两个亚群，CD8^+^CD28^+^是具有细胞毒性的T淋巴细胞，而CD8^+^CD28^-^是具有免疫抑制作用的调节性T细胞。本研究发现CD8在正常组、癌前病变组、原发性肺癌组、淋巴结转移性肺癌组的阳性率逐步增高，原发性肺癌组与正常组的差异有统计学意义（*P* < 0.05），提示机体发生肿瘤时，CD8^+^CD28^-^调节T细胞免疫抑制活性增强，肿瘤免疫中T细胞介导的抗肿瘤作用降低。我们研究结果显示Ⅲ期+Ⅳ期患者CD8的阳性率低于Ⅰ期+Ⅱ期（*P* < 0.05），其原因可能为个体差异。CD8^+^CD28^+^亚群与肿瘤直接接触，通过释放穿孔素和颗粒酶等细胞毒性物质，溶解靶细胞，也可能通过释放一些细胞因子诱导靶细胞的凋亡，其机制有待于进一步研究。

CTLA-4属CD28家族成员，是重要的T细胞免疫负性调节因子。其生物学特性使其可通过多种可能途径影响肿瘤的发生和发展。CTLA-4主要表达于活化的T细胞表面，与B7分子结合可抑制T细胞活化和增殖，从而降低抗肿瘤免疫效应。早期研究^[9]^证实CTLA-4拮抗剂可降低动物模型中的肿瘤发生率和肿瘤分级。近年Ribas等^[10]^给予进展期转移性黑色素瘤患者CTLA-4抗体，结果显示其可激活免疫系统，且在部分患者中观察到持久且客观的抗肿瘤免疫应答反应。我们的研究发现CTLA-4在癌细胞和间质淋巴细胞中均有表达，在正常组、癌前病变组、原发性肺癌组、淋巴结转移性肺癌组的阳性率逐渐增高，原发性肺癌组与正常组之间、癌前病变组与正常组之间差异均具有统计学意义（*P* < 0.05），说明CTLA-4可能不仅作为免疫调节分子发挥作用，可能对细胞有其它的生物学功能，肿瘤细胞中CTLA-4的高表达可能会改变肿瘤免疫微环境活化与抑制的平衡，使肿瘤逃避免疫系统的监控。本研究显示CTLA-4在年龄≥60岁组阳性率高于 < 60岁组（*P* < 0.05），低+未分化组高于高+中分化组（*P* < 0.05），随肿瘤进展和分化程度降低，CTLA-4表达阳性率逐渐增高，提示CTLA-4的表达与年龄、分化程度有关。

我们研究还发现原发性肺癌中gp96、CD8、CTLA-4三者表达密切相关，gp96与CTLA-4在肺癌组织中的表达呈正相关，与CD8的表达呈负相关，CD8与CTLA-4的表达呈负相关（*P* < 0.05）。说明gp96作为分子伴侣参与了肿瘤抗原向MHC-I类分子途径的递呈过程，并与抗原肽形成gp96-肽复合物激活CD8^+^T淋巴细胞，产生抗肿瘤的特异性免疫反应; CTLA-4可以通过抑制IL-2的生成，从而减少CD8^+^T淋巴细胞数量，抑制抗肿瘤免疫反应; gp96和CTLA-4可能是通过某种途径共同作用于树突状细胞，发挥其抗原递呈功能，进而诱导肿瘤的特异免疫，有关机制还有待于进一步研究。
